# Serum 25-hydroxyvitamin D, systemic immune-inflammation index, and atherogenic index of plasma in statin-untreated type 2 diabetes: a cross-sectional mediation analysis

**DOI:** 10.3389/fcvm.2026.1884708

**Published:** 2026-10-05

**Authors:** Hüseyin Avni Fındıklı, Vehbi Şirikçi

**Affiliations:** Department of Internal Medicine, Kahramanmaraş Necip Fazıl City Hospital, Kahramanmaraş, Türkiye

**Keywords:** 25-hydroxyvitamin D, atherogenic index of plasma, mediation analysis, non-linear relationship, statin-untreated, systemic immune-inflammation index, type 2 diabetes

## Abstract

This cross-sectional study investigated the associations between serum 25-hydroxyvitamin D [25(OH)D] levels, systemic inflammatory burden, and atherogenic lipid profile in statin-untreated adults with type 2 diabetes, with particular emphasis on the contribution of the systemic immune-inflammation index (SII). A total of 619 statin-untreated patients with type 2 diabetes were included. Participants were stratified according to serum 25(OH)D concentrations into deficient (<20 ng/mL), insufficient (20–29.9 ng/mL), and sufficient (≥30 ng/mL) groups. Multivariable linear regression, BCa bootstrap mediation analysis with 5,000 resamples, and restricted cubic spline models were used. In fully adjusted models, each 10 ng/mL decrease in serum 25(OH)D was independently associated with higher SII (β=0.064, 95% CI 0.023–0.105; *p* = 0.002) and higher atherogenic index of plasma (AIP; β=0.031, 95% CI 0.009–0.053; *p* = 0.006). The indirect association through SII accounted for 26.5% of the total association between 25(OH)D and AIP (bootstrap 95% CI 12.8–43.1). Indirect associations were weaker for the neutrophil-to-lymphocyte ratio and non-significant for the monocyte-to-lymphocyte and platelet-to-lymphocyte ratios. Restricted cubic spline analyses demonstrated significant non-linearity for both SII and AIP, with points of maximal curvature at 18.7 ng/mL and 19.6 ng/mL, respectively. Estimates were similar in overlapping laboratory subgroups, but the indirect association was not significant in men (19.2%, 95% CI −7.6 to 39.4) or after exclusion of patients with severe deficiency (<10 ng/mL), although formal testing showed no significant interaction with sex. In a sensitivity analysis for unmeasured mediator–outcome confounding, the indirect association was reduced to zero at a residual correlation of only *ρ*=0.20, indicating limited robustness. The findings are compatible with a statistical indirect relationship involving systemic inflammation between vitamin D status and atherogenic dyslipidaemia, but the cross-sectional design precludes causal inference. The non-linear pattern near 20 ng/mL suggests that the inflammatory–atherogenic phenotype may become more pronounced below this threshold.

## 1 Introduction

Type 2 diabetes is a complex cardiometabolic phenotype characterised by the interplay of chronic low-grade systemic inflammation, insulin resistance, and atherogenic dyslipidaemia (1, 2). In this setting, accumulation of triglyceride-rich remnant lipoproteins, impaired HDL function, and small, dense LDL particles are major drivers of atherosclerotic cardiovascular disease (3, 4). Composite indices such as the atherogenic index of plasma (AIP), the logarithmic form of the triglyceride-to-HDL-C ratio, therefore provide a more comprehensive assessment of atherogenic lipid burden than conventional lipid parameters (5, 6).

Vitamin D insufficiency is common in type 2 diabetes and is associated with insulin resistance, endothelial dysfunction, and heightened cardiovascular risk (7, 8). Through the vitamin D receptor (VDR), its active form modulates NF-*κ*B and MAPK pathways, suppressing pro-inflammatory cytokine production and influencing lipoprotein metabolism (9, 10). However, the extent to which the association between low serum 25-hydroxyvitamin D [25(OH)D] levels and atherogenic dyslipidaemia operates through inflammatory pathways remains unclear.

The systemic immune-inflammation index (SII), which integrates neutrophil, platelet, and lymphocyte counts, has emerged as a robust and easily measurable marker of systemic inflammation with strong prognostic value in cardiovascular and metabolic diseases (11, 12). Nevertheless, its potential role as an intermediate variable in the relationship between 25(OH)D insufficiency and atherogenic dyslipidaemia has not been well studied. Most prior studies have focused on the general population, inadequately accounted for statin effects, and lacked comprehensive quantitative mediation and non-linear analyses.

In the present study, we investigated the associations between serum 25(OH)D levels, systemic inflammatory burden, and atherogenic lipid profile in statin-untreated adults with type 2 diabetes. We particularly examined the indirect association through SII in the relationship between 25(OH)D and AIP, compared with alternative haematologic indices (NLR, MLR, and PLR), and evaluated the non-linear shape of these associations using restricted cubic spline analyses.

## 2 Methods

### 2.1 Study design and ethical approval

This retrospective, cross-sectional study evaluated the associations between serum 25-hydroxyvitamin D [25(OH)D] levels, systemic inflammation, and atherogenic lipid profiles in statin-untreated adults with type 2 diabetes. De-identified data were retrieved from electronic medical records of the Internal Medicine clinics at Kahramanmaraş Necip Fazıl City Hospital. The study protocol was approved by the Medical Research Ethics Committee of Kahramanmaraş Sütçü İmam University (Decision No:330, Date: 01.09.25) and conducted in accordance with the Declaration of Helsinki. The institutional ethics committee waived the requirement for written informed consent due to the retrospective design and use of fully anonymized data.

### 2.2 Study population and patient selection

Adult patients with type 2 diabetes presenting between September 1, 2023, and September 1, 2025, were screened. To avoid confounding from the pleiotropic effects of lipid-lowering agents, the cohort was restricted to statin-untreated patients, defined as no statin or lipid-lowering therapy within the preceding three months. Eligibility was operationalised at the index laboratory measurement as the absence of both a hyperlipidaemia diagnosis code and any statin or other lipid-lowering prescription in the preceding three months; the cohort therefore reflects a three-month treatment-free window rather than lifetime non-exposure. Of 4,788 screened patients, 619 met the eligibility criteria and were included in the final analysis (Figure 1). Of the 4,169 patients excluded, 2,862 lacked complete or concurrent laboratory measurements, 1,040 were receiving statin or other lipid-lowering therapy, and 267 met one of the remaining clinical exclusion criteria. Eligible patients were aged 18–65 years with a diagnosis of type 2 diabetes recorded as an ICD diagnosis code in the electronic medical record, and with concurrent laboratory measurements for serum 25(OH)D, HbA1c, lipid profile, and complete blood count. Measurements were required to have been drawn within one month of each other; this window reflects routine practice in the outpatient diabetes clinics, where these tests belong to a single follow-up episode but are not invariably drawn on the same day. Exclusion criteria were leukocytosis (>11,000/µL), chronic systemic inflammatory or autoimmune diseases, hepatic failure, active or past malignancy, pregnancy, vitamin D supplementation within the past three months, or missing laboratory data. Anaemia and reduced kidney function were not applied as exclusion criteria; haemoglobin and eGFR were instead included as covariates in the multivariable models and were additionally examined in sensitivity analyses restricted to non-anaemic patients and to patients with eGFR ≥60 mL/min/1.73 m^2^.

**Figure 1:**
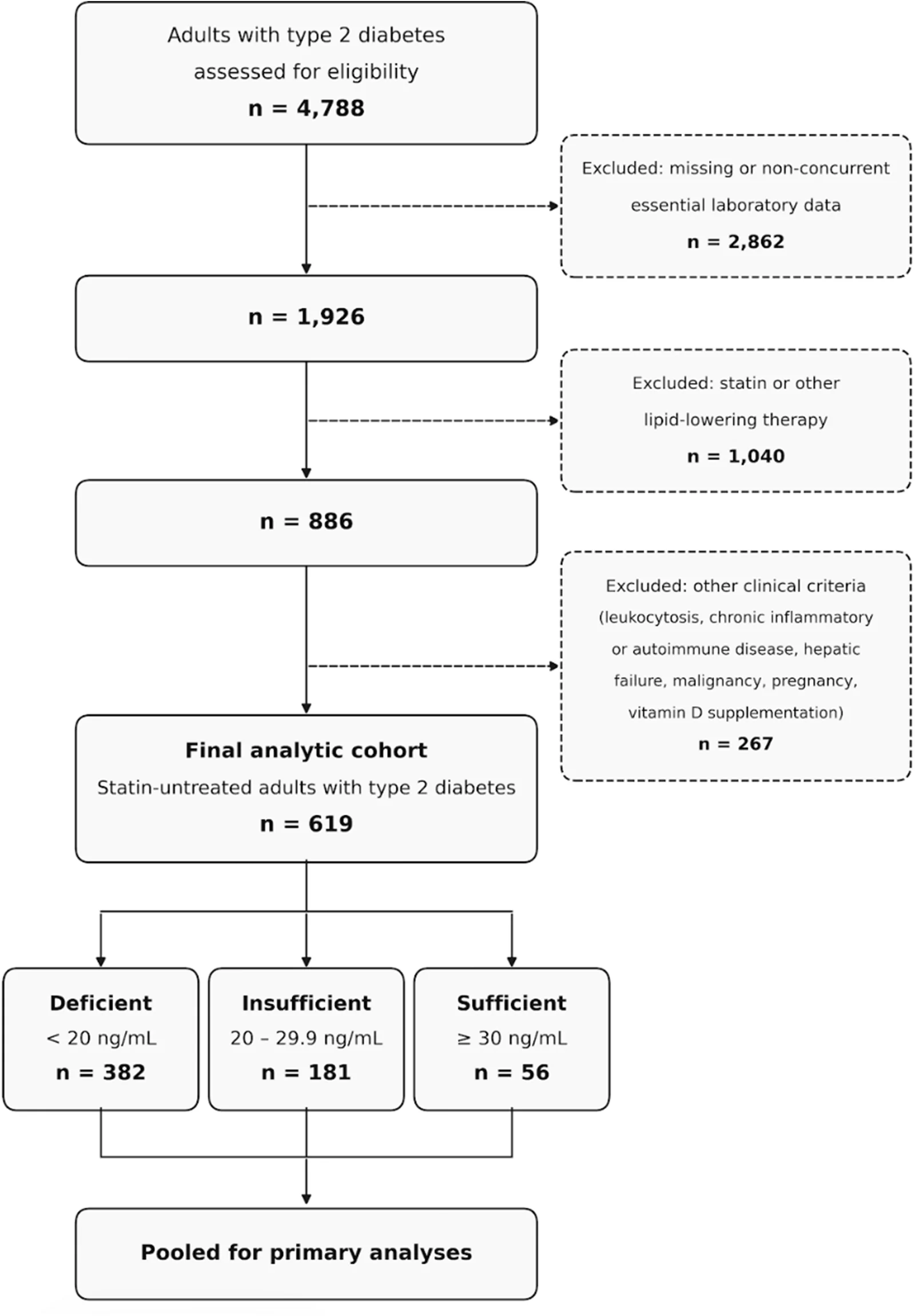
Selection of the statin-untreated type 2 diabetes cohort and stratification by serum 25(OH)D status. Flowchart showing the derivation of the final analytic cohort from adults with type 2 diabetes screened between September 1, 2023, and September 1, 2025. Patients were excluded according to predefined criteria, including statin or other lipid-lowering therapy use, missing or non-concurrent key laboratory data, vitamin D supplementation within the preceding three months, leukocytosis (>11,000/µL), hepatic failure, active or past malignancy, pregnancy, and chronic systemic inflammatory or autoimmune disease. Anaemia and reduced kidney function were not applied as exclusion criteria; haemoglobin and eGFR were handled as covariates in the multivariable models and as sensitivity-analysis restrictions. The final cohort was stratified according to serum 25(OH)D status as deficient (<20 ng/mL), insufficient (20–29.9 ng/mL), or sufficient (≥30 ng/mL). 25(OH)D, 25-hydroxyvitamin D.

### 2.3 Data collection, 25(OH)D categorisation, and derived indices

Demographic, clinical, and laboratory parameters—including age, sex, HbA1c, serum 25(OH)D, a complete lipid panel, and full blood counts—were extracted from electronic medical records. Estimated glomerular filtration rate (eGFR) was calculated using the 2021 CKD-EPI race-free equation (13). To control for seasonal variation, the month of 25(OH)D measurement was categorized into winter (December–February), spring (March–May), summer (June–August), and autumn (September–November). All assays were performed using standard methods in the hospital central laboratory. Lipid panels are ordered after an overnight fast as routine practice in these clinics; because the data were collected retrospectively, adherence to fasting could not be verified for individual patients. Serum 25(OH)D was measured by chemiluminescence immunoassay on a Mindray CL-8000i analyser (Shenzhen Mindray Bio-Medical Electronics Co., Ltd., Shenzhen, China). Manufacturer-reported coefficients of variation were 2.79–3.39% within-run and 3.27%–3.80% within-device. Established cardiovascular disease was ascertained from diagnosis codes recorded in the electronic medical records and was defined as a documented history of coronary artery disease, cerebrovascular events, or peripheral artery disease; patients with established cardiovascular disease were not excluded, and this variable is reported as a baseline characteristic.

Serum 25(OH)D levels were categorized per Endocrine Society guidelines into deficiency (<20 ng/mL), insufficiency (20–29.9 ng/mL), and sufficiency (≥30 ng/mL), and were also modeled as a continuous variable (7, 14). The primary inflammatory marker, systemic immune-inflammation index (SII), was calculated as (platelets×neutrophils)/lymphocytes. Alternative hematologic indices included neutrophil-to-lymphocyte (NLR), monocyte-to-lymphocyte (MLR), and platelet-to-lymphocyte (PLR) ratios. The primary lipid outcome was the atherogenic index of plasma [AIP=log₁₀(triglycerides/HDL-C)], with both triglyceride and HDL-C concentrations expressed in mmol/L in accordance with the original definition of the index. Supporting indices included remnant cholesterol (total cholesterol – LDL-C – HDL-C) and non-HDL cholesterol (total cholesterol – HDL-C). The TG/HDL-C ratio was not analysed as a separate outcome because it is a monotone transformation of AIP; it is reported only as a descriptive characteristic in the baseline table.

### 2.4 Statistical analysis

The normality of continuous variables was assessed using the Shapiro–Wilk test and visual methods, including Q–Q plots and histograms. Data were presented as mean ± standard deviation or median with interquartile range (IQR), according to distributional characteristics. Categorical variables were expressed as counts and percentages. Between-group comparisons were performed using one-way ANOVA or the Kruskal–Wallis test, as appropriate, whereas categorical variables were compared using Pearson's chi-square test or Fisher's exact test. SII, NLR, MLR, and remnant cholesterol were natural log-transformed [ln(x)] before regression analyses; AIP, PLR, and non-HDL-C were modeled on their original scales. Patients with missing values for any of the index laboratory variables were not eligible, so the analytic cohort was complete for the exposure, mediator and outcome; missingness in the remaining covariates was below 5% and complete-case analysis was applied.

Independent associations between serum 25(OH)D levels and inflammatory or lipid indices were examined using four hierarchical multivariable linear regression models: Model 1, unadjusted; Model 2, adjusted for age and sex; Model 3, adjusted for Model 2 covariates plus HbA1c and eGFR; and Model 4, fully adjusted for Model 3 covariates plus ALT, hemoglobin, WBC, and season. Covariates were selected *a priori* on clinical grounds and from prior literature rather than by data-driven selection, and were entered in blocks so that the contribution of each is visible: demographic determinants of both vitamin D status and lipid profile (age, sex); the principal metabolic and renal determinants of 25(OH)D metabolism and atherogenic lipids (HbA1c, eGFR); and hepatic, haematologic and seasonal factors (ALT, haemoglobin, WBC, season of measurement), season being a well-established determinant of circulating 25(OH)D. Multicollinearity was assessed using the variance inflation factor, with VIF <5 considered acceptable. Non-linear patterns between serum 25(OH)D levels and SII or AIP were evaluated using restricted cubic spline (RCS) models based on the Model 4 covariate set. Four knots were placed at the 5th, 35th, 65th, and 95th percentiles of the 25(OH)D distribution, with 20 ng/mL specified as the reference value. RCS analyses were performed using the rms package. Points of maximal curvature were derived *post hoc* from the fitted spline functions by numerical differentiation. For each outcome the fitted curve was evaluated on a fine grid spanning the observed range of serum 25(OH)D, and the point of maximal curvature was defined as the concentration maximising the geometric curvature function *κ*(x) = |f″(x)|/[1 + f′(x)^2^]^{3/2}^. These points were estimated only for outcomes with a significant test for non-linearity and are interpreted as descriptive features of the fitted curves rather than prespecified clinical thresholds. Because geometric curvature depends on the scaling of both axes, the values are specific to the units used here [25(OH)D in ng/mL; the outcome on its modelled scale] and should not be read as scale-invariant thresholds. They are also distinct from points of inflection, which are defined by f″(x) = 0.

The mediating role of SII in the association between 25(OH)D and AIP was evaluated using causal mediation analysis within a counterfactual framework. Because the design is cross-sectional, the sequential ignorability assumption underlying counterfactual mediation cannot be verified; the estimated indirect effect is therefore interpreted as a statistical indirect association rather than a causal mediation effect. Total, direct and indirect effects were estimated separately, together with the proportion mediated, calculated as (indirect effect/total effect) × 100 and interpreted as a descriptive statistical quantity rather than a causal quantity. Inference for the indirect effect was based on 95% confidence intervals (CIs) obtained using bias-corrected and accelerated (BCa) bootstrap resampling with 5,000 iterations. Mediation analyses were performed using the mediation package. NLR, MLR, and PLR were tested as alternative mediators, while remnant cholesterol and non-HDL cholesterol were evaluated as alternative outcome variables. The stability of the main findings was assessed by repeating the mediation model in laboratory subgroups (WBC ≤10.0 × 10^3^/µL, non-anaemic patients, eGFR ≥60 mL/min/1.73 m^2^, ALT within the normal range), in clinical subgroups defined by sex, and after excluding patients with severe vitamin D deficiency (<10 ng/mL). Because a difference in statistical significance between strata is not evidence of a difference in effect, effect modification by sex was assessed formally in addition to reporting stratum-specific estimates. Restricted cubic spline models were additionally fitted separately in women and men and in strata defined by a median split of HbA1c, with knots fixed at the percentiles of the whole-cohort 25(OH)D distribution so that curve shape could be compared on a common parameterisation; 25(OH)D × sex and 25(OH)D × HbA1c-stratum interaction terms were tested in the pooled spline models, and differences in the indirect effect between sexes were evaluated using a test of moderated mediation. Sensitivity of the indirect effect to unmeasured mediator–outcome confounding was quantified by varying the correlation (*ρ*) between the residuals of the mediator and outcome models and identifying the value at which the estimated indirect effect reached zero, together with the corresponding product of the proportions of residual variance explained by an unmeasured confounder (R̃^2^_M_ × R̃^2^_Y_, which equals *ρ*^2^ in this parameterisation).

Data preprocessing, cleaning, and diagnostic visualizations were performed in Python (v3.11). Hierarchical regression, RCS modeling, and mediation analyses were conducted in R (v4.3.2). All statistical tests were two-sided, and *p* < 0.05 was considered statistically significant. No adjustment for multiple comparisons was applied; AIP and SII were the principal outcome and mediator of interest, and findings for the remaining haematologic and lipid indices should be regarded as exploratory.

## 3 Results

### 3.1 Cohort derivation and baseline phenotype across serum 25(OH)D categories

During the study period, 4,788 patients with type 2 diabetes were retrospectively screened. After applying predefined inclusion and exclusion criteria, 619 statin-untreated patients were included in the final analytic cohort. Participants were categorized according to serum 25-hydroxyvitamin D [25(OH)D] status as deficient (<20 ng/mL; *n* = 382), insufficient (20–29.9 ng/mL; *n* = 181), or sufficient (≥30 ng/mL; *n* = 56) (Figure 1). The median age of the cohort was 49.0 years (IQR 40.0–58.0), and 64.0% were female. Age, sex distribution, HbA1c, creatinine, eGFR, ALT, haemoglobin, lymphocyte count, monocyte count, MLR, PLR, total cholesterol, LDL-C, and seasonal distribution did not differ significantly across 25(OH)D categories (all *p* > 0.05). Established cardiovascular disease was documented in 32 patients (5.2%). Median LDL-C was 124 mg/dL (IQR 102–145); 491 patients (79.3%) had LDL-C ≥ 100 mg/dL, 263 (42.5%) ≥ 130 mg/dL and 97 (15.7%) ≥ 160 mg/dL. Among the 1,926 screened patients with complete concurrent laboratory data, 1,040 (54.0%) were receiving lipid-lowering therapy and were excluded; established cardiovascular disease was documented in 261 (25.1%) of them.

In contrast, several inflammatory and atherogenic parameters differed significantly across groups. White blood cell count decreased from the deficient to the sufficient group (median 8.2, 7.9, and 7.7 × 10^3^/µL, respectively; *p* = 0.041), as did neutrophil count (4.8, 4.5, and 4.3 × 10^3^/µL; *p* = 0.038). Correspondingly, the neutrophil-to-lymphocyte ratio (NLR) was 2.39, 2.12, and 1.96 (*p* = 0.016), and the systemic immune-inflammation index (SII) was 675, 575, and 520 (*p* = 0.011) across the deficient, insufficient, and sufficient groups.

A similar pattern was observed for atherogenic lipid measures. HDL-C levels were lower in the deficient group (47, 50, and 51 mg/dL; *p* = 0.026), whereas triglycerides (164, 146, and 136 mg/dL; *p* = 0.018), non-HDL-C (160, 150, and 145 mg/dL; *p* = 0.044), and remnant cholesterol (34, 29, and 27 mg/dL; *p* = 0.021) were higher. Differences were more evident for composite atherogenic indices: AIP was 0.17, 0.09, and 0.04 (*p* = 0.006), and the TG/HDL-C ratio was 3.36, 2.80, and 2.52 (*p* = 0.006) across the three groups, respectively (Table 1; Figure 2).

**Table 1:** Baseline seasonal, inflammatory, and atherogenic characteristics according to serum 25(OH)D categories in statin-untreated patients with type 2 diabetes.

Variable	Total (*n* = 619)	Deficient <20 ng/mL (*n* = 382)	Insufficient 20–29.9 ng/mL (*n* = 181)	Sufficient ≥30 ng/mL (*n* = 56)	*p-*value
Age, years	49.0 (40.0–58.0)	50.0 (41.0–59.0)	48.0 (39.0–57.0)	47.0 (38.0–56.0)	0.118
Female, *n* (%)	396 (64.0)	253 (66.2)	111 (61.3)	32 (57.1)	0.238
HbA1c, %	8.1 (7.1–9.3)	8.2 (7.2–9.5)	8.0 (7.0–9.1)	7.8 (6.9–8.9)	0.089
Creatinine, mg/dL	0.81 (0.70–0.92)	0.82 (0.71–0.94)	0.80 (0.69–0.91)	0.79 (0.68–0.90)	0.284
eGFR	102 (91–113)	101 (89–112)	103 (92–114)	104 (94–115)	0.224
ALT, U/L	24 (17–35)	25 (18–37)	23 (17–34)	22 (16–32)	0.083
25(OH)D, ng/mL	16.9 (9.9–23.5)	10.7 (8.6–15.8)	23.7 (21.5–26.4)	32.6 (30.8–35.7)	<0.001
Season, *n* (%)					0.074
Winter, *n* (%)	164 (26.5)	110 (28.8)	43 (23.8)	11 (19.6)	
Spring, *n* (%)	168 (27.1)	108 (28.3)	47 (26.0)	13 (23.2)	
Summer, *n* (%)	143 (23.1)	78 (20.4)	47 (26.0)	18 (32.1)	
Autumn, *n* (%)	144 (23.3)	86 (22.5)	44 (24.3)	14 (25.0)	
WBC, ×10^3^/µL	8.1 (6.8–9.3)	8.2 (6.9–9.5)	7.9 (6.7–9.2)	7.7 (6.5–8.9)	0.041
Hemoglobin, g/dL	13.8 (12.6–15.0)	13.7 (12.5–15.0)	13.9 (12.8–15.1)	14.0 (12.8–15.2)	0.248
Platelet, ×10^3^/µL	278 (239–326)	279 (240–327)	277 (238–325)	276 (236–323)	0.670
Neutrophil, ×10^3^/µL	4.7 (3.7–5.8)	4.8 (3.8–5.9)	4.5 (3.6–5.6)	4.3 (3.4–5.3)	0.038
Lymphocyte, ×10^3^/µL	2.1 (1.7–2.6)	2.0 (1.6–2.5)	2.2 (1.8–2.7)	2.2 (1.8–2.8)	0.074
Monocyte, ×10^3^/µL	0.55 (0.43–0.68)	0.56 (0.44–0.70)	0.53 (0.42–0.66)	0.52 (0.40–0.64)	0.092
NLR	2.26 (1.64–3.10)	2.39 (1.75–3.28)	2.12 (1.55–2.92)	1.96 (1.45–2.68)	0.016
MLR	0.26 (0.19–0.34)	0.27 (0.20–0.35)	0.24 (0.18–0.32)	0.23 (0.17–0.31)	0.064
PLR	132 (92–192)	140 (96–204)	126 (88–181)	125 (84–179)	0.094
SII	632 (435–925)	675 (468–982)	575 (399–830)	520 (360–755)	0.011
TC, mg/dL	204 (177–232)	208 (181–238)	201 (174–228)	197 (170–224)	0.072
LDL-C, mg/dL	124 (102–145)	126 (104–148)	122 (100–141)	119 (97–137)	0.118
HDL-C, mg/dL	49 (42–56)	47 (40–55)	50 (43–57)	51 (44–59)	0.026
TG, mg/dL	154 (110–221)	164 (118–236)	146 (104–206)	136 (98–192)	0.018
Non-HDL-C, mg/dL	154 (130–185)	160 (135–189)	150 (126–179)	145 (121–173)	0.044
Remnant-C, mg/dL	30 (21–46)	34 (23–50)	29 (20–43)	27 (18–39)	0.021
AIP	0.13 (−0.05–0.30)	0.17 (−0.01–0.35)	0.09 (−0.08–0.25)	0.04 (−0.13–0.22)	0.006
TG/HDL-C	3.08 (2.05–4.58)	3.36 (2.24–5.12)	2.80 (1.90–4.06)	2.52 (1.68–3.78)	0.006

Data are presented as median (IQR) or *n* (%). *p* values were calculated using the Kruskal–Wallis test for continuous variables and Pearson's *χ*^2^ test for categorical variables; Fisher's exact test was used when appropriate. Season was analyzed as a single categorical variable, and one overall *p* value is reported. Severe vitamin D deficiency was defined as serum 25(OH)D < 10 ng/mL and was present in 156 patients; these patients were included within the deficient category and excluded only in the corresponding sensitivity analysis. 25(OH)D, 25-hydroxyvitamin D; AIP, atherogenic index of plasma; ALT, alanine aminotransferase; eGFR, estimated glomerular filtration rate (mL/min/1.73 m^2^); HbA1c, glycated hemoglobin; HDL-C, high-density lipoprotein cholesterol; LDL-C, low-density lipoprotein cholesterol; MLR, monocyte-to-lymphocyte ratio; NLR, neutrophil-to-lymphocyte ratio; PLR, platelet-to-lymphocyte ratio; SII, systemic immune-inflammation index; TC, total cholesterol; TG, triglyceride; WBC, white blood cell count. AIP was calculated from molar (mmol/L) concentrations of triglycerides and HDL-C. The TG/HDL-C ratio is shown on the conventional mg/dL scale; because it is a monotone transformation of AIP it shares the same Kruskal–Wallis *p* value and was not analysed as a separate outcome.

**Figure 2:**
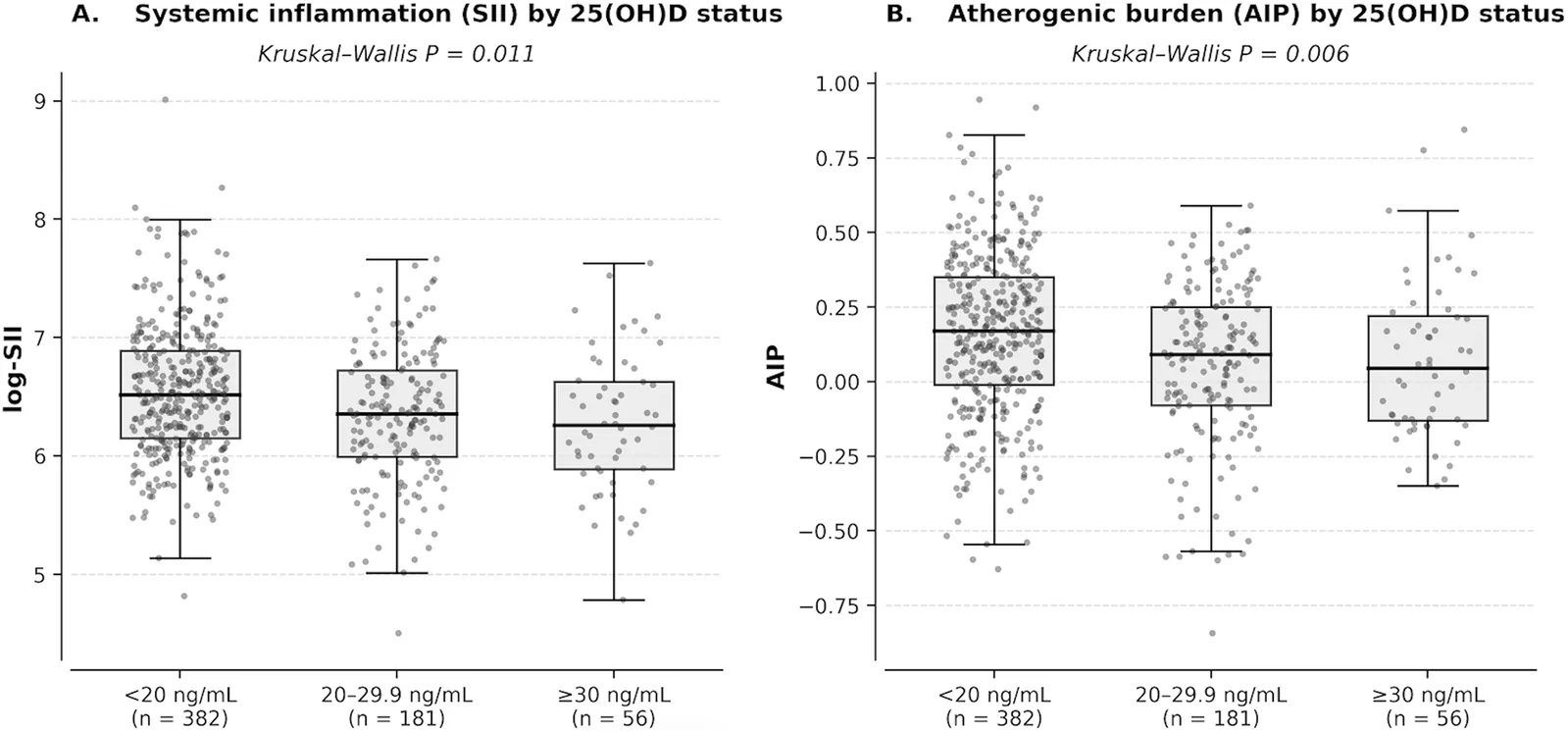
Systemic inflammation **(A)** and atherogenic lipid burden **(B)** across serum 25(OH)D categories. Boxplots show the median (horizontal line), the interquartile range (box) and 1.5×IQR whiskers; individual observations are displayed as jittered points. Group comparisons were performed using Kruskal–Wallis tests. SII was log-transformed before visualization because of its skewed distribution. AIP was calculated as log₁₀(TG/HDL-C), with both concentrations expressed in mmol/L, and was plotted without additional transformation. SII, systemic immune-inflammation index; AIP, atherogenic index of plasma; 25(OH)D, 25-hydroxyvitamin D.

### 3.2 Multivariable evidence linking lower 25(OH)D to SII and AIP

The independent associations of serum 25(OH)D with inflammatory and atherogenic indices were examined using hierarchical linear regression models, with effects expressed per 10 ng/mL decrement in 25(OH)D. In unadjusted models, lower 25(OH)D levels were significantly associated with AIP, remnant cholesterol, and non-HDL-C; the strongest association among lipid indices was observed for AIP (β=0.049; 95% CI 0.027–0.071; *p* < 0.001). Among inflammatory indices, lower 25(OH)D was associated with SII (β=0.096; 95% CI 0.054–0.138; *p* < 0.001) and NLR (β=0.056; 95% CI 0.017–0.095; *p* = 0.005).

After full adjustment for age, sex, HbA1c, eGFR, ALT, haemoglobin, WBC, and season of measurement, the associations became more selective. Each 10 ng/mL decrease in 25(OH)D remained independently associated with higher AIP (β=0.031; 95% CI 0.009–0.053; *p* = 0.006) and higher SII (β=0.064; 95% CI 0.023–0.105; *p* = 0.002). Associations with remnant cholesterol, non-HDL-C, NLR, MLR, and PLR were no longer statistically significant in fully adjusted models (Table 2). Intermediate models and adjusted R^2^ values are provided in Supplementary Table S1.

**Table 2:** Multivariable associations of serum 25(OH)D with atherogenic lipid burden and hematologic inflammatory indices.

Outcome	Predictor	Model 1 (unadjusted)		Model 4 (fully adjusted)	
		β (95% CI)	*p*	β (95% CI)	*p*
Atherogenic lipid burden					
AIP	Per 10 ng/mL ↓	0.049 (0.027–0.071)	<0.001	0.031 (0.009–0.053)	0.006
Remnant-C*	Per 10 ng/mL ↓	0.047 (0.012–0.082)	0.008	0.024 (−0.010–0.058)	0.166
Non-HDL-C	Per 10 ng/mL ↓	4.1 (0.7–7.5)	0.019	2.0 (−1.1–5.1)	0.204
Hematologic inflammatory burden					
SII*	Per 10 ng/mL ↓	0.096 (0.054–0.138)	<0.001	0.064 (0.023–0.105)	0.002
NLR*	Per 10 ng/mL ↓	0.056 (0.017–0.095)	0.005	0.032 (−0.005–0.069)	0.089
MLR*	Per 10 ng/mL ↓	0.031 (−0.003–0.065)	0.074	0.016 (−0.017–0.049)	0.338
PLR	Per 10 ng/mL ↓	0.019 (−0.015–0.053)	0.273	0.007 (−0.026–0.040)	0.677

Linear regression coefficients (β) express the change in each outcome per 10 ng/mL decrease in serum 25(OH)D. Model 1 was unadjusted. Model 4 was adjusted for age, sex, HbA1c, eGFR, ALT, hemoglobin, WBC, and season of 25(OH)D measurement. *Log-transformed before regression. AIP, PLR, and non-HDL-C were modeled on their original scales. All variance inflation factor values were <5, indicating no relevant multicollinearity. 25(OH)D, 25-hydroxyvitamin D; AIP, atherogenic index of plasma; ALT, alanine aminotransferase; CI, confidence interval; eGFR, estimated glomerular filtration rate; HbA1c, glycated hemoglobin A1c; HDL-C, high-density lipoprotein cholesterol; MLR, monocyte-to-lymphocyte ratio; NLR, neutrophil-to-lymphocyte ratio; non-HDL-C, non–high-density lipoprotein cholesterol; PLR, platelet-to-lymphocyte ratio; Remnant-C, remnant cholesterol; SII, systemic immune-inflammation index; WBC, white blood cell count.

### 3.3 SII as the principal indirect pathway in the 25(OH)D–AIP association

Counterfactual mediation analyses with BCa bootstrap resampling (5,000 iterations) were performed to quantify the indirect association between 25(OH)D and AIP through each haematologic inflammatory index. In the primary model, the indirect association through SII was statistically significant. For each 10 ng/mL decrease in 25(OH)D, the total effect (c) was 0.031, the direct effect (c′) was 0.023, and the indirect effect through SII (a × b) was 0.0082. The indirect effect was statistically significant (95% CI 0.0021–0.0154; *p* = 0.006), with the indirect association through SII accounting for 26.5% of the total association (95% CI 12.8–43.1).

In comparative analyses of candidate mediators, the indirect association through NLR was weaker but statistically significant (16.5%; 95% CI 2.1–31.6; *p* = 0.038), whereas indirect associations through MLR and PLR were not significant. When alternative atherogenic outcomes were examined, the indirect association was not significant for remnant cholesterol or non-HDL-C. This pattern indicates that the indirect signal was concentrated on AIP (Table 3; Figure 3).

**Table 3:** Indirect associations between serum 25(OH)D and atherogenic indices: comparison of candidate inflammatory mediators and outcome variables.

Mediator	Outcome	Total effect (c)	Direct effect (c′)	Indirect effect (a × b)	95% CI (indirect)	Proportion mediated, % (95% CI)	*p*
Comparison of inflammatory mediators (outcome: AIP)							
SII	AIP	**0** **.** **031**	**0** **.** **023**	**0** **.** **0082**	**0.0021–0.0154**	**26.5** **(12.8–43.1)**	**0** **.** **006**
NLR	AIP	0.031	0.026	0.0051	0.0004–0.0118	16.5 (2.1–31.6)	0.038
MLR	AIP	0.031	0.028	0.0030	−0.0024–0.0091	9.7 (−7.2–25.4)	0.214
PLR	AIP	0.031	0.030	0.0014	−0.0031–0.0068	4.5 (−9.8–18.7)	0.486
SII as mediator across atherogenic outcomes							
SII	Remnant-C	0.024	0.020	0.0042	−0.0014–0.0112	17.5 (−5.3–34.8)	0.126
SII	Non-HDL-C	2.0	1.7	0.3	−0.4–0.9	15.0 (−10.4–32.6)	0.392

Exposure was serum 25(OH)D, modeled per 10 ng/mL decrease. Total, direct, and indirect effects were estimated using counterfactual mediation analysis; proportion mediated was calculated as (indirect effect/total effect) × 100. Confidence intervals and two-sided *p* values were derived from BCa bootstrap resampling with 5,000 iterations. All models were adjusted for age, sex, HbA1c, eGFR, ALT, hemoglobin, WBC, and season of 25(OH)D measurement. AIP is log-transformed by definition [AIP=log₁₀(TG/HDL-C)]. SII, NLR, MLR, and remnant cholesterol were log-transformed before analysis; PLR and non-HDL-C were modeled on their original scales. Effects for non-HDL-C are expressed in mg/dL; effects for transformed variables are expressed on the log scale. The indirect association was considered significant when the 95% CI for the indirect effect excluded zero. Bold values indicate the primary mediation model (SII as mediator, AIP as outcome).

**Figure 3:**
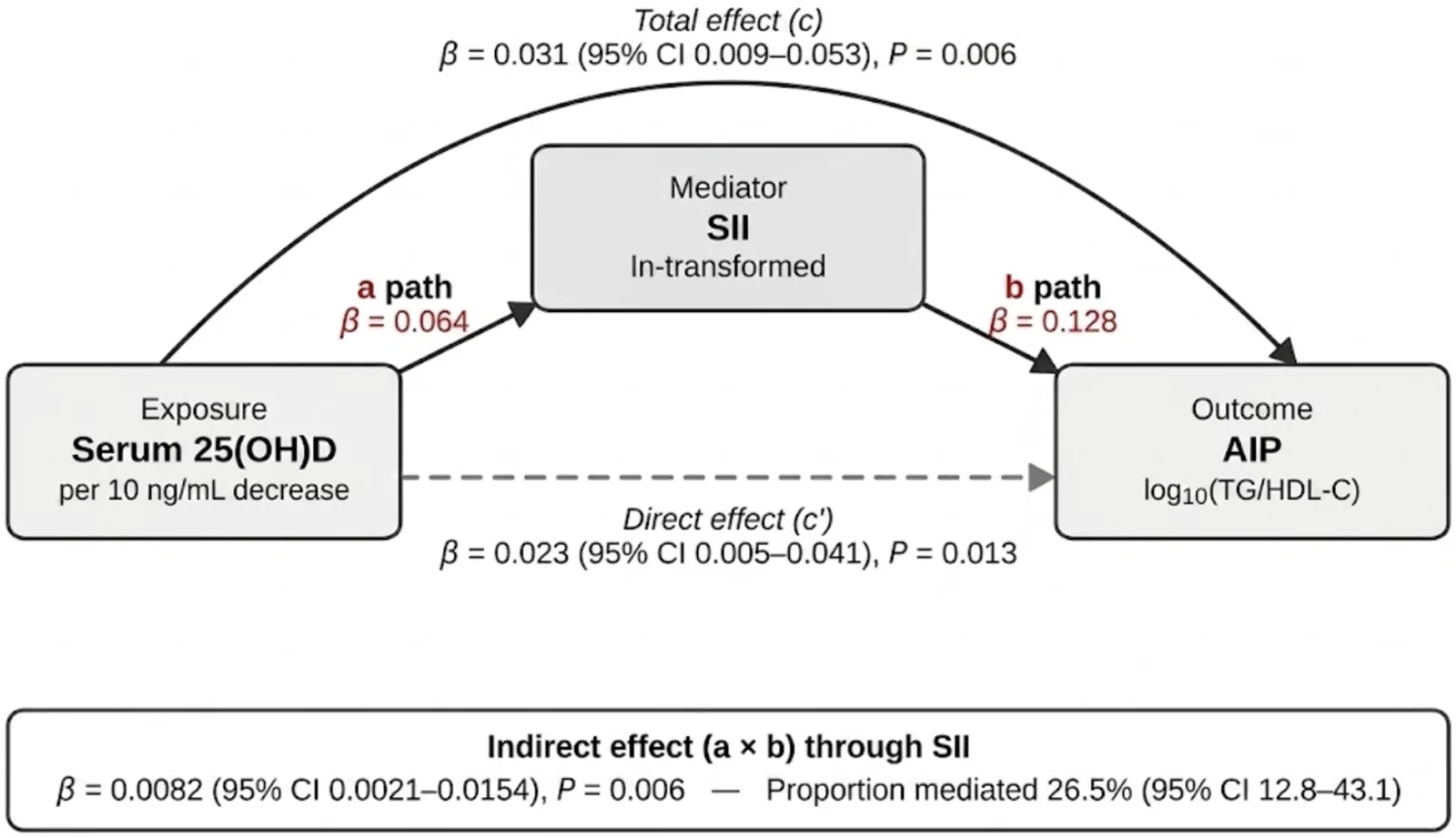
Indirect association between serum 25(OH)D and atherogenic lipid burden through SII. Counterfactual mediation model evaluating the indirect association through SII between serum 25(OH)D, modeled per 10 ng/mL decrease, and AIP. Solid arrows depict the indirect pathway through SII (a × b); the dashed arc represents the direct effect (c′); and the upper arc represents the total effect (c). Effects were estimated using BCa bootstrap resampling with 5,000 iterations, with statistical significance inferred when the 95% confidence interval for the indirect effect excluded zero. Models were adjusted for age, sex, HbA1c, eGFR, ALT, hemoglobin, WBC, and season of 25(OH)D measurement. SII was log-transformed before modeling, whereas AIP was modeled on its original scale. 25(OH)D, 25-hydroxyvitamin D; AIP, atherogenic index of plasma; CI, confidence interval; SII, systemic immune-inflammation index.

### 3.4 Non-linear associations of serum 25(OH)D with SII and AIP

Restricted cubic spline models anchored at 20 ng/mL, the Endocrine Society deficiency threshold, were used to examine potential non-linear relationships. Knots were placed at the 5th, 35th, 65th, and 95th percentiles of the serum 25(OH)D distribution, and all models were fully adjusted. Significant non-linear associations were observed for SII and AIP. For SII, the overall association was significant (*p* overall=0.004), as was the test for non-linearity (p non-linearity=0.018), with the point of maximal curvature at 18.7 ng/mL. For AIP, the overall association was also significant (p overall=0.014), with evidence of non-linearity (p non-linearity=0.047) and a point of maximal curvature at 19.6 ng/mL.

Relative to the 20 ng/mL reference, adjusted β coefficients for SII were 0.041 (95% CI 0.014–0.068) at 15 ng/mL and 0.091 (95% CI 0.038–0.144) at 10 ng/mL. Corresponding estimates for AIP were 0.018 (95% CI 0.002–0.034) and 0.041 (95% CI 0.008–0.074), respectively. No significant non-linearity was observed for NLR, MLR, PLR, remnant cholesterol, or non-HDL-C. The non-linear pattern was therefore most evident along the SII–AIP axis, with both points of maximal curvature clustering near the 20 ng/mL threshold (Table 4; Figure 4).

**Table 4:** Restricted cubic spline analysis of non-linear associations within the 25(OH)D–inflammation–atherogenicity axis.

Outcome	*p* (overall)	*p* (non-linearity)	Point of maximal curvature	β (95% CI) at 15 ng/mL	β (95% CI) at 10 ng/mL
SII*	**0** **.** **004**	**0** **.** **018**	**18.7 ng/mL**	**0.041** **(0.014–0.068)**	**0.091** **(0.038–0.144)**
AIP	**0** **.** **014**	**0** **.** **047**	**19.6 ng/mL**	**0.018** **(0.002–0.034)**	**0.041** **(0.008–0.074)**
NLR*	0.071	0.112	—	0.019 (−0.006–0.044)	0.044 (−0.004–0.092)
Remnant-C*	0.132	0.164	—	0.014 (−0.008–0.036)	0.030 (−0.012–0.072)
Non-HDL-C	0.219	0.238	—	1.0 (−0.7–2.7)	2.2 (−1.1–5.5)
MLR*	0.301	0.241	—	0.010 (−0.008–0.028)	0.024 (−0.012–0.060)
PLR	0.594	0.356	—	0.004 (−0.014–0.022)	0.010 (−0.026–0.046)

Restricted cubic spline models were fitted with knots placed at the 5th, 35th, 65th, and 95th percentiles of serum 25(OH)D and anchored at 20 ng/mL, the conventional Endocrine Society deficiency threshold. β coefficients represent adjusted differences relative to the 20 ng/mL reference value, where β = 0. Estimates are shown at 15 ng/mL and 10 ng/mL as clinically relevant values within the deficient range. Effects for non-HDL-C are expressed in mg/dL; effects for log-transformed variables are expressed on the log scale. Models were adjusted for age, sex, HbA1c, eGFR, ALT, hemoglobin, WBC, and season of 25(OH)D measurement. Points of maximal curvature were estimated only when the test for non-linearity was significant and are descriptive features of the fitted curves rather than clinical cutoffs. They are not points of inflection in the mathematical sense, which would require f″(x) = 0. *Log-transformed before spline modeling. AIP, PLR, and non-HDL-C were modeled on their original scales. 25(OH)D, 25-hydroxyvitamin D; AIP, atherogenic index of plasma; ALT, alanine aminotransferase; CI, confidence interval; eGFR, estimated glomerular filtration rate; HbA1c, glycated hemoglobin A1c; MLR, monocyte-to-lymphocyte ratio; NLR, neutrophil-to-lymphocyte ratio; non-HDL-C, non–high-density lipoprotein cholesterol; PLR, platelet-to-lymphocyte ratio; Remnant-C, remnant cholesterol; SII, systemic immune-inflammation index; WBC, white blood cell count. Bold values indicate outcomes with a statistically significant test for non-linearity (*p* < 0.05).

**Figure 4:**
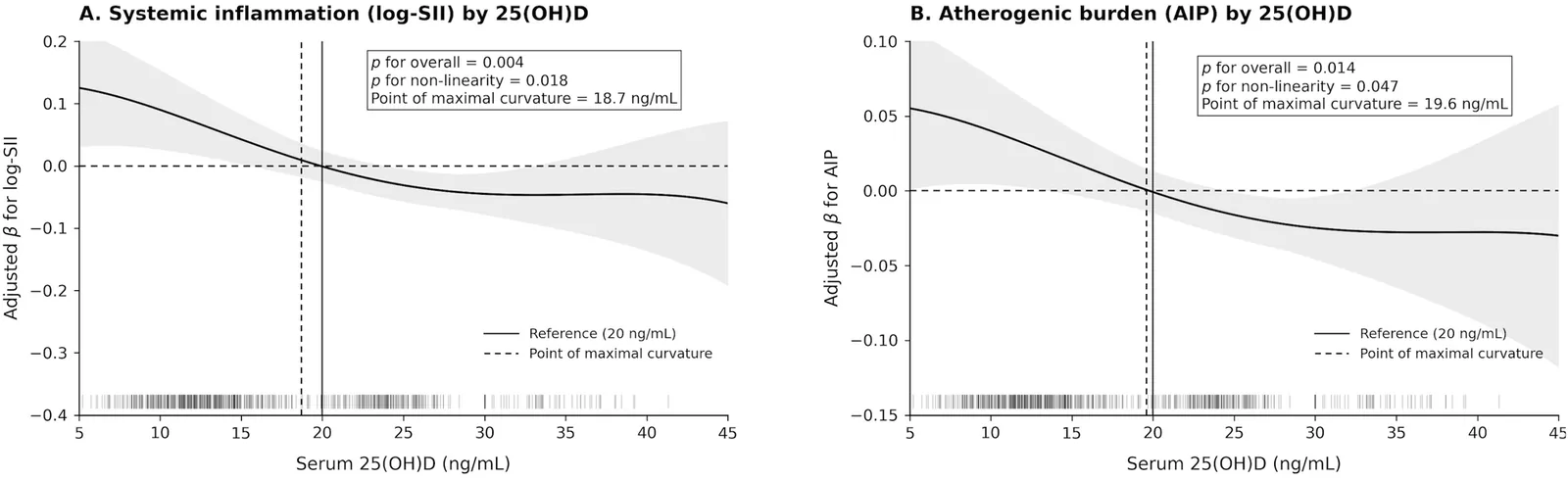
Non-linear associations of serum 25(OH)D with systemic inflammation and atherogenic lipid burden. Restricted cubic spline models show adjusted associations between serum 25(OH)D and **(A)** SII or **(B)** AIP; SII was log-transformed before modeling, whereas AIP was modeled on its original scale. Solid lines represent adjusted β coefficients, and shaded areas indicate 95% confidence intervals. The solid vertical line denotes the 20 ng/mL reference value; the dashed vertical line denotes the point of maximal curvature. Models were adjusted for age, sex, HbA1c, eGFR, ALT, hemoglobin, WBC, and season of 25(OH)D measurement. Knots were placed at the 5th, 35th, 65th, and 95th percentiles of serum 25(OH)D. Wider confidence intervals at higher 25(OH)D levels reflect lower sample density. SII, systemic immune-inflammation index; AIP, atherogenic index of plasma; 25(OH)D, 25-hydroxyvitamin D.

### 3.5 Sensitivity and subgroup analyses of the indirect association through SII

Sensitivity and subgroup analyses were conducted to examine how the indirect association through SII between 25(OH)D and AIP behaved across clinical and laboratory subsets of the cohort. The proportion mediated observed in the main cohort (26.5%) remained largely consistent across laboratory subgroups: 24.5% (95% CI 10.4–40.6; *p* = 0.014) in patients with WBC ≤10.0 × 10^3^/µL, 25.3% (95% CI 11.5–41.9; *p* = 0.011) in non-anaemic patients, 25.2% (95% CI 11.9–41.2; *p* = 0.009) in those with eGFR ≥60 mL/min/1.73 m^2^, and 22.6% (95% CI 5.8–38.7; *p* = 0.031) in patients with ALT within the normal range. In each of these subgroups the indirect effect through SII remained statistically significant; these subsets retained 82%–96% of the analytic cohort, however, so their consistency reflects limited change in sample composition rather than independent confirmation. In clinical subgroups, the proportion mediated was 28.1% (95% CI 13.5–46.8; *p* = 0.008) in female patients, whereas it was lower and non-significant in male patients (19.2%; 95% CI −7.6–39.4; *p* = 0.154). When patients with severe vitamin D deficiency (<10 ng/mL) were excluded, the proportion mediated decreased to 20.8% and lost statistical significance (95% CI −3.4–38.9; *p* = 0.081). These analyses indicate that the indirect association through SII was preserved across the laboratory subgroups but attenuated after exclusion of severe vitamin D deficiency and in male patients (Table 5; Figure 5). Formal testing provided no evidence that the difference between sexes reflected true effect modification. The 25(OH)D × sex interaction was not significant for either SII (*p* = 0.31; non-linear component *p* = 0.44) or AIP (*p* = 0.39; non-linear component *p* = 0.52), and the difference in the indirect effect between sexes was not significant in a test of moderated mediation (*p* = 0.28). In sex-stratified spline models the association was weaker and less precise in men (SII: p overall=0.118, p non-linearity=0.284; AIP: p overall=0.246, p non-linearity=0.411) than in women (SII: p overall=0.004, p non-linearity=0.032; AIP: p overall=0.021, p non-linearity=0.061), while the subgroup confidence intervals for the proportion mediated overlapped extensively. Spline models stratified by glycaemic control showed a comparable pattern: the association was significant in patients with HbA1c below the cohort median of 8.1% (*n* = 309; SII p overall=0.011, p non-linearity=0.043; AIP p overall=0.019) and not significant in those at or above it (*n* = 310; SII p overall=0.081; AIP p overall=0.128), while the 25(OH)D × HbA1c interaction was not significant for either outcome (SII *p* = 0.146, non-linear component *p* = 0.228; AIP *p* = 0.214, non-linear component *p* = 0.317). The two strata were of similar size, so the difference in stratum-specific significance is unlikely to be explained by a marked difference in precision; the formal test nevertheless provides no evidence of effect modification, and the association was in no case stronger in the stratum with poorer glycaemic control (Supplementary Table S2). In the sensitivity analysis for unmeasured mediator–outcome confounding, the estimated indirect effect reached zero when the correlation between the residuals of the mediator and outcome models was *ρ*=0.20, corresponding to R̃^2^_M_ × R̃^2^_Y_ = 0.041; this is a modest threshold and indicates limited robustness of the indirect association to unmeasured mediator–outcome confounding.

**Table 5:** Sensitivity and subgroup analyses of the indirect association through SII between 25(OH)D and AIP.

Analysis set	*n*	Total effect (c)	Direct effect (c′)	Indirect effect (a × b)	95% CI (indirect)	Proportion mediated, % (95% CI)	*p*
Main cohort — reference	**619**	**0** **.** **031**	**0** **.** **023**	**0** **.** **0082**	**0.0021–0.0154**	**26.5** **(12.8–43.1)**	**0** **.** **006**
Sensitivity by laboratory subgroup							
WBC ≤ 10.0 × 10^3^/*μ*L	548	0.029	0.022	0.0071	0.0015–0.0140	24.5 (10.4–40.6)	0.014
Non-anemic subgroup	531	0.030	0.023	0.0076	0.0018–0.0148	25.3 (11.5–41.9)	0.011
eGFR ≥ 60 mL/min/1.73 m^2^	594	0.031	0.024	0.0078	0.0019–0.0149	25.2 (11.9–41.2)	0.009
ALT within normal range	506	0.027	0.021	0.0061	0.0008–0.0127	22.6 (5.8–38.7)	0.031
Sensitivity by clinical subgroup							
Excluding severe deficiency (< 10 ng/mL)	463	0.025	0.020	0.0052	−0.0007–0.0114	20.8 (−3.4–38.9)	0.081
Female patients	396	0.032	0.023	0.0090	0.0023–0.0176	28.1 (13.5–46.8)	0.008
Male patients	223	0.026	0.021	0.0050	−0.0021–0.0129	19.2 (−7.6–39.4)	0.154

Exposure: serum 25(OH)D, modeled per 10 ng/mL decrease. Mediator: log-transformed SII. Outcome: AIP. Proportion mediated was calculated as (indirect effect/total effect) × 100. Confidence intervals and two-sided *p* values were derived from BCa bootstrap resampling with 5,000 iterations within each subgroup. All models were adjusted for age, sex, HbA1c, eGFR, ALT, hemoglobin, WBC, and season of 25(OH)D measurement, except when the covariate served as the stratifying variable. The indirect association was considered significant when the 95% CI for the indirect effect excluded zero. The non-anemic subgroup was defined using sex-specific hemoglobin thresholds: ≥12 g/dL for females and ≥13 g/dL for males. 25(OH)D, 25-hydroxyvitamin D; AIP, atherogenic index of plasma; ALT, alanine aminotransferase; CI, confidence interval; eGFR, estimated glomerular filtration rate; HbA1c, glycated hemoglobin A1c; SII, systemic immune-inflammation index; WBC, white blood cell count. Bold values indicate the main-cohort reference estimate.

**Figure 5:**
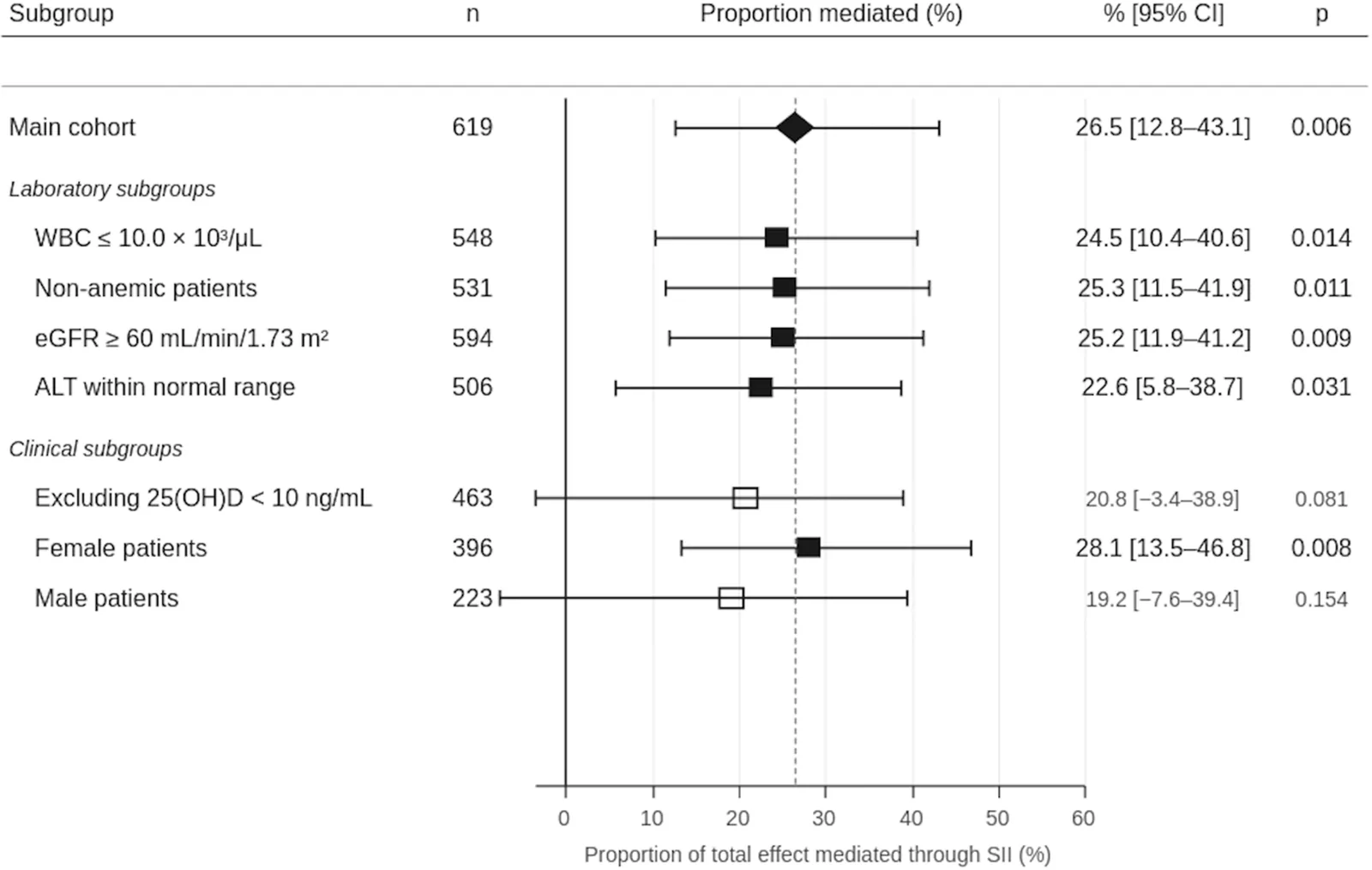
Sensitivity and subgroup analyses of the indirect association through SII between 25(OH)D and AIP. Forest plot showing the proportion of the total association between serum 25(OH)D, modeled per 10 ng/mL decrease, and AIP mediated through SII across the main cohort and seven sensitivity subgroups. The diamond represents the main cohort estimate, and squares represent subgroup estimates. Filled markers indicate a statistically significant indirect association, defined by a 95% confidence interval excluding zero; open markers indicate a non-significant indirect association. Horizontal lines represent 95% confidence intervals derived from BCa bootstrap resampling with 5,000 iterations within each subgroup. The solid vertical line denotes the null effect, and the dashed vertical line marks the main cohort estimate of 26.5%. Models were adjusted for age, sex, HbA1c, eGFR, ALT, hemoglobin, WBC, and season of 25(OH)D measurement, except when the covariate served as the stratifying variable. 25(OH)D, 25-hydroxyvitamin D; AIP, atherogenic index of plasma; ALT, alanine aminotransferase; CI, confidence interval; eGFR, estimated glomerular filtration rate; HbA1c, glycated hemoglobin A1c; SII, systemic immune-inflammation index; WBC, white blood cell count.

## 4 Discussion

In this cross-sectional study of statin-untreated adults with type 2 diabetes, lower serum 25-hydroxyvitamin D [25(OH)D] levels were independently associated with increased systemic inflammatory burden and a more atherogenic lipid profile. These associations were more evident with composite indices that integrate inflammation and atherogenic dyslipidaemia — particularly the atherogenic index of plasma (AIP) and the systemic immune-inflammation index (SII) — than with conventional lipid parameters. The indirect association through SII accounted for 26.5% (bootstrap 95% CI 12.8–43.1) of the total association between lower 25(OH)D and AIP. This is compatible with a statistical indirect relationship involving systemic inflammation between vitamin D status and atherogenic lipid dysregulation, although the cross-sectional design does not allow a biological pathway to be established. The indirect association was strongest for SII, weaker but still significant for the neutrophil-to-lymphocyte ratio, and absent for the monocyte-to-lymphocyte and platelet-to-lymphocyte ratios. This gradient is consistent with a neutrophil–lymphocyte signal rather than a non-specific haematological shift; platelet count and PLR showed no association with 25(OH)D in any analysis, so the platelet component of SII does not appear to contribute to the observed pattern. Why SII nonetheless showed a stronger and more stable association than NLR is uncertain and may reflect differences in distribution and residual variance between the two indices rather than additional biological information. Restricted cubic spline analyses further revealed points of maximal curvature for SII and AIP at 18.7 ng/mL and 19.6 ng/mL, respectively — close to the conventional 20 ng/mL threshold, so that the points of maximal curvature for both outcomes cluster in the same narrow range. We are not aware of a previous quantitative mediation analysis of the 25(OH)D–SII–AIP axis in a statin-untreated type 2 diabetes cohort that also characterises the non-linear shape of these associations.

Our findings should be interpreted within the broader literature examining the relationships between 25(OH)D, systemic inflammation, and atherogenic lipid metabolism. Large population-based studies have demonstrated independent associations between lower 25(OH)D levels and both inflammatory markers and atherogenic lipids (5, 8). The association between SII and AIP has itself been described in the general population (15); what has not been quantified is the extent to which it lies between 25(OH)D and AIP. However, studies integrating these three components within a single quantitative mediation framework remain limited, and results have been inconsistent. For instance, Lhilali et al. reported associations between vitamin D insufficiency and atherogenic lipid indices in healthy women but did not examine inflammatory intermediates (16), while Lin et al. reported no mediating effect of SII in the relationship between 25(OH)D and respiratory dysfunction in over 17,000 participants (12). These discrepancies suggest that the pathways linking 25(OH)D to downstream metabolic outcomes are context-dependent, varying according to clinical setting, metabolic risk profile, and the inflammatory marker employed. By focusing on a well-defined statin-untreated type 2 diabetes population where inflammation and atherogenic dyslipidaemia coexist, the present study addresses an important gap in the literature.

The indirect association through SII was statistically significant in this statin-untreated cohort (26.5%; 95% CI 12.8–43.1). This result does not contradict the negative mediation signal reported by Lin et al. in the general population; rather, it supports the notion that the 25(OH)D–inflammation relationship is highly sensitive to the underlying metabolic context. In clinical phenotypes such as type 2 diabetes, where chronic low-grade inflammation and dyslipidaemia coexist, inflammatory signals that may be masked in heterogeneous populations become more apparent. Whether composite cell-based indices such as SII capture vitamin D-related subclinical inflammation better than single acute-phase reactants could not be tested here, because hsCRP is not routinely measured in this setting. This pattern was reinforced by the absence of an indirect association through MLR and PLR, although the significant indirect association through NLR indicates that the signal is not unique to SII. Consistent convergence was also observed in non-linear analyses, with the points of maximal curvature for both SII and AIP clustering near 20 ng/mL. This positions approximately 20 ng/mL as an exploratory concentration region in statin-untreated type 2 diabetes, in the sense that the points of maximal curvature of both fitted curves fall close to it.

The pattern of indirect association we observed is biologically coherent with the known cellular and molecular effects of 25(OH)D on inflammation and lipid metabolism. The active form of vitamin D, 1,25-dihydroxyvitamin D, acts via the vitamin D receptor (VDR) expressed on monocytes, macrophages, and T-lymphocytes to inhibit NF-*κ*B and MAPK signalling, thereby suppressing pro-inflammatory cytokines (TNF-α, IL-6, IL-1β) while enhancing anti-inflammatory IL-10 responses (9, 17). *In vitro* evidence confirms that 25(OH)D₃ and 1,25(OH)₂D₃ attenuate lipopolysaccharide-induced TNF-α expression and NF-*κ*B activation in macrophages (18). At the endothelial level, vitamin D can limit pro-atherothrombotic activation by reducing IL-6-driven tissue factor and adhesion molecule expression (10). In this mechanistic context, vitamin D insufficiency may promote chronic low-grade inflammation, neutrophil activation, impaired regulatory T-cell function, and enhanced platelet–leucocyte interactions — all processes well captured by SII. The associations we observed between low 25(OH)D, elevated SII and higher AIP are consistent with these experimental observations, although the present design cannot demonstrate that the same mechanisms operate in this cohort.

Biologically consistent links also exist between 25(OH)D status and lipoprotein metabolism. Huang et al. demonstrated that higher 25(OH)D levels were positively associated with lipoprotein lipase (LPL) activity, which in turn was inversely related to insulin resistance and type 2 diabetes (19). Vitamin D insufficiency has additionally been linked to upregulated SREBP-1c expression, increased hepatic triglyceride synthesis, and VLDL secretion. Systemic inflammation may exacerbate this imbalance by suppressing LPL activity and impairing HDL's anti-atherogenic and anti-inflammatory functions (20, 21). In type 2 diabetes, hyperglycaemia further compromises HDL functionality, creating a vicious cycle between inflammation and atherogenesis (22). The finding that approximately one quarter of the 25(OH)D–AIP association was indirect through SII is compatible with crosstalk between immuno-inflammatory and LPL–VLDL–TG/HDL-C pathways. Sensitivity analyses showed a consistent indirect association across the laboratory subgroups, with attenuation after exclusion of severe deficiency (<10 ng/mL) and in the smaller male subgroup; the former is consistent with the non-linear pattern observed in the spline models. This should be viewed as a hypothesis-generating signal rather than a definitive therapeutic threshold. Two subgroup findings deserve explicit interpretation. First, the proportion mediated differed between women (28.1%, 95% CI 13.5–46.8) and men (19.2%, 95% CI −7.6–39.4), but formal testing provided no evidence of effect modification: the 25(OH)D × sex interaction was not significant for SII (*p* = 0.31) or AIP (*p* = 0.39), the difference in the indirect association between sexes was not significant in a test of moderated mediation (*p* = 0.28), and the subgroup confidence intervals overlapped extensively. A difference in statistical significance between strata is not evidence of a difference in effect, and the pattern observed here is compatible with the smaller male subgroup (*n* = 223) and its wider uncertainty rather than with a sex-specific mechanism. Second, excluding patients with severe deficiency (<10 ng/mL) reduced the proportion mediated to 20.8% and removed statistical significance. This exclusion removes about one quarter of the cohort (*n* = 156) together with the lowest end of the exposure distribution, which is precisely the range in which the spline models indicate the association steepens; both the total association (0.031 to 0.025) and the indirect effect estimate (0.0082 to 0.0052) attenuated alongside the loss of precision, so magnitude and certainty were affected together. Because the indirect association could not be demonstrated above 10 ng/mL, these findings should not be read as evidence for an inflammatory pathway within the 10–20 ng/mL range. This pattern is internally consistent with the non-linear findings, but it should be acknowledged that the indirect association was not independently confirmed outside the severely deficient range.

The key clinical implication of our findings is that in statin-untreated patients with type 2 diabetes, low 25(OH)D, and in particular concentrations below the 20 ng/mL deficiency threshold, marks a phenotype in which subclinical inflammation and atherogenic dyslipidaemia occur together. Low 25(OH)D in this population co-occurred with higher systemic inflammation and a heavier atherogenic lipid burden. Whether the combination of 25(OH)D, SII and AIP adds prognostic information beyond established risk factors was not tested: no cardiovascular outcomes were available, AIP is a surrogate lipid index rather than a clinical endpoint, and no discrimination or reclassification analysis was performed. These cross-sectional data therefore do not prove that SII is a causal therapeutic target, that the triad identifies a high-risk group, or that vitamin D supplementation will improve lipid indices.

Our focus on a statin-untreated cohort is both a strength and a limitation. Statins exert pleiotropic effects beyond LDL-C reduction, including improvement of endothelial function, suppression of pro-inflammatory cytokines, and modulation of platelet and neutrophil activity (23–25). By studying statin-untreated patients, we captured a more unconfounded inflammatory–atherogenic profile. While this enhances internal validity, it may limit generalisability, as most patients with type 2 diabetes in clinical practice receive statins. The cohort should not, however, be read as a low-risk phenotype selected out of treatment: established cardiovascular disease was documented in only 5.2%, compared with 25.1% of those excluded for lipid-lowering therapy, yet 79.3% had LDL-C ≥ 100 mg/dL and 42.5% ≥ 130 mg/dL. The cohort is therefore largely a primary-prevention population with substantial residual LDL-C burden rather than one in which treatment was not indicated, and the findings are most directly applicable to that group. Eligibility also required the absence of a recorded hyperlipidaemia diagnosis code, so patients whose dyslipidaemia had been recognised but not treated were not included; the cohort is therefore one in which dyslipidaemia was neither coded nor treated, which should be borne in mind when generalising. Future studies should evaluate how this axis behaves in statin-treated populations. The strengths of this study include a clinically homogeneous, statin-untreated cohort restricted to adults aged 18–65 years, which minimised confounding from lipid-lowering therapy and from age-related immuno-metabolic alterations (such as inflammaging, sarcopenia, and renal–hepatic functional decline) that typically obscure vitamin D–inflammation–lipid relationships; comprehensive covariate adjustment; comparative evaluation of multiple inflammatory indices; and the combined use of multivariable regression, bootstrap mediation, and restricted cubic spline modelling. Several limitations should be noted. The design is cross-sectional and single-centre, so temporality cannot be established and reverse causality is plausible: serum 25(OH)D behaves as a negative acute-phase reactant and falls during inflammatory states (26), so a low concentration may partly reflect inflammatory burden rather than precede it. Spline models stratified by glycaemic control did not show the association concentrated in patients with higher HbA1c, but the interaction test was null in both directions and HbA1c is at best an indirect proxy for the acute-phase state that lowers circulating 25(OH)D. This analysis therefore does not adjudicate the direction of the association, and reverse causality remains a plausible explanation. Body mass index is not recorded in the hospital information system, and fasting insulin, HOMA-IR and hsCRP are not routinely measured, so no proxy for adiposity or insulin resistance could be modelled. Smoking status, alcohol consumption, dietary intake, physical activity, sun exposure and duration of diabetes are likewise not captured in the electronic record. Antidiabetic medication was outside the scope of the approved data extraction, so the distribution of metformin, insulin, GLP-1 receptor agonists and SGLT2 inhibitors across 25(OH)D categories could neither be described nor adjusted for, although these agents may influence body weight, systemic inflammation and lipid metabolism. Each of these variables plausibly influences both circulating 25(OH)D and the inflammatory–atherogenic phenotype, so residual confounding cannot be excluded; consistent with this, the indirect association was reduced to zero at a residual mediator–outcome correlation of only *ρ*=0.20, a modest threshold indicating limited robustness. Serum 25(OH)D and SII were each measured at a single time point, and although overt leukocytosis was excluded, subclinical infection, physiological stress and corticosteroid exposure cannot be ruled out; a single measurement cannot establish the temporal stability of either variable. Fasting is routine practice for lipid testing in these clinics but could not be verified retrospectively, so occasional non-fasting triglyceride values, and hence some misclassification of AIP, cannot be excluded. Exposure, mediator and outcome were not always measured on the same day, which adds measurement noise to the estimated indirect association. eGFR was included as a covariate, but residual confounding from mildly reduced kidney function remains possible. The cohort was also deliberately restricted to adults aged 18–65 years, which limits direct extrapolation to older patients. The points of maximal curvature near 20 ng/mL should be considered exploratory and require validation in prospective cohorts.

## 5 Conclusions

In conclusion, lower serum 25-hydroxyvitamin D [25(OH)D] levels were independently associated with increased systemic inflammation and a more atherogenic lipid profile in statin-untreated adults with type 2 diabetes. The indirect association through the systemic immune-inflammation index (SII) accounted for approximately one-quarter (26.5%) of the association between lower 25(OH)D and the atherogenic index of plasma (AIP). This is compatible with a statistical indirect relationship involving systemic inflammation between vitamin D status and atherogenic dyslipidaemia, although the cross-sectional design, the attenuation seen in sensitivity analyses, and the limited robustness to unmeasured mediator–outcome confounding preclude a causal interpretation. Restricted cubic spline analyses further identified points of maximal curvature near 20 ng/mL for both outcomes, suggesting that the inflammatory–atherogenic phenotype may become more pronounced below this concentration. Whether the combined evaluation of 25(OH)D, SII and AIP carries prognostic value in statin-untreated patients with type 2 diabetes was not tested here and requires prospective evaluation. Prospective cohort studies and interventional trials are warranted to establish causality, define clinically meaningful thresholds, and determine whether vitamin D supplementation or inflammation-targeted strategies can modify this pathway.

## Data Availability

The raw data supporting the conclusions of this article will be made available by the authors, without undue reservation.
