# Supplementary material for: Serum 25-hydroxyvitamin D, systemic immune-inflammation index, and atherogenic index of plasma in statin-untreated type 2 diabetes: a cross-sectional mediation analysis
Source: Front Cardiovasc Med. 2026 Oct 5;13:1884708. doi: 10.3389/fcvm.2026.1884708 (PMC13677915; doi:10.3389/fcvm.2026.1884708)
Supplement: Supplementary file 1 [file Table1.docx]

**Supplementary Table S1.** Stepwise multivariable associations of serum 25(OH)D with atherogenic lipid burden and hematologic inflammatory indices, with model performance metrics.

| **Outcome** | **Model 1** | | **Model 2** | | **Model 3** | | **Model 4** | | **Adj. R²** |
| --- | --- | --- | --- | --- | --- | --- | --- | --- | --- |
|  | **β (95% CI)** | ***p*** | **β (95% CI)** | ***p*** | **β (95% CI)** | ***p*** | **β (95% CI)** | ***p*** | **(Model 4)** |
| ***Atherogenic lipid burden*** | | | | | | | | | |
| AIP | 0.049 (0.027–0.071) | <0.001 | 0.045 (0.023–0.067) | <0.001 | 0.036 (0.014–0.058) | 0.002 | 0.031 (0.009–0.053) | 0.006 | 0.16 |
| Remnant-C* | 0.047 (0.012–0.082) | 0.008 | 0.041 (0.006–0.076) | 0.022 | 0.029 (−0.005–0.063) | 0.094 | 0.024 (−0.010–0.058) | 0.166 | 0.11 |
| Non-HDL-C | 4.1 (0.7–7.5) | 0.019 | 3.5 (0.1–6.9) | 0.044 | 2.5 (−0.7–5.7) | 0.124 | 2.0 (−1.1–5.1) | 0.204 | 0.10 |
| ***Hematologic inflammatory burden*** | | | | | | | | | |
| SII* | 0.096 (0.054–0.138) | <0.001 | 0.089 (0.047–0.131) | <0.001 | 0.075 (0.034–0.116) | <0.001 | 0.064 (0.023–0.105) | 0.002 | 0.18 |
| NLR* | 0.056 (0.017–0.095) | 0.005 | 0.050 (0.011–0.089) | 0.012 | 0.039 (0.001–0.077) | 0.045 | 0.032 (−0.005–0.069) | 0.089 | 0.12 |
| MLR* | 0.031 (−0.003–0.065) | 0.074 | 0.027 (−0.007–0.061) | 0.119 | 0.021 (−0.012–0.054) | 0.212 | 0.016 (−0.017–0.049) | 0.338 | 0.07 |
| PLR | 0.019 (−0.015–0.053) | 0.273 | 0.016 (−0.018–0.050) | 0.356 | 0.011 (−0.022–0.044) | 0.514 | 0.007 (−0.026–0.040) | 0.677 | 0.05 |

Linear regression coefficients (β) express the change in each outcome per 10 ng/mL decrease in serum 25(OH)D. Model 1: unadjusted. Model 2: adjusted for age and sex. Model 3: Model 2 plus HbA1c and eGFR. Model 4: Model 3 plus ALT, hemoglobin, WBC, and season of 25(OH)D measurement. Adjusted R² values refer to the fully adjusted Model 4. *Log-transformed prior to regression. AIP, PLR, and non-HDL-C were modeled on their original scales. Abbreviations: 25(OH)D, 25-hydroxyvitamin D; AIP, atherogenic index of plasma; ALT, alanine aminotransferase; CI, confidence interval; eGFR, estimated glomerular filtration rate; HbA1c, glycated hemoglobin A1c; HDL-C, high-density lipoprotein cholesterol; MLR, monocyte-to-lymphocyte ratio; NLR, neutrophil-to-lymphocyte ratio; non-HDL-C, non–high-density lipoprotein cholesterol; PLR, platelet-to-lymphocyte ratio; Remnant-C, remnant cholesterol; SII, systemic immune-inflammation index; WBC, white blood cell count.

**Supplementary Table S2.** Subgroup-stratified restricted cubic spline analysis of serum 25(OH)D with SII and AIP.

| **Subgroup** | **Outcome** | **p (overall)** | **p (non-linearity)** | **Point of maximal curvature** |
| --- | --- | --- | --- | --- |
| **Panel A — by sex** | | | | |
| Women (n = 396) | SII* | 0.004 | 0.032 | 18.5 ng/mL |
| Men (n = 223) | SII* | 0.118 | 0.284 | — |
| Women (n = 396) | AIP | 0.021 | 0.061 | — |
| Men (n = 223) | AIP | 0.246 | 0.411 | — |
| **Panel B — by glycaemic control** | | | | |
| HbA1c < 8.1% (n = 309) | SII* | 0.011 | 0.043 | 18.9 ng/mL |
| HbA1c ≥ 8.1% (n = 310) | SII* | 0.081 | 0.176 | — |
| HbA1c < 8.1% (n = 309) | AIP | 0.019 | 0.072 | — |
| HbA1c ≥ 8.1% (n = 310) | AIP | 0.128 | 0.263 | — |

Models were fitted separately within each stratum using the Model 4 covariate set, with knots fixed at the 5th, 35th, 65th and 95th percentiles of the whole-cohort serum 25(OH)D distribution so that curve shape is compared on a common parameterisation. Strata for glycaemic control were defined by a median split of HbA1c (8.1%), chosen to preserve comparable power in both strata. Points of maximal curvature were estimated only where the test for non-linearity was significant. Stratum-specific estimates are descriptive. For sex, the 25(OH)D × sex interaction was not significant for SII (p = 0.31; non-linear component p = 0.44) or AIP (p = 0.39; non-linear component p = 0.52), and the difference in the indirect effect between sexes was not significant in a test of moderated mediation (p = 0.28). For glycaemic control, the 25(OH)D × HbA1c interaction was not significant for SII (p = 0.146; non-linear component p = 0.228) or AIP (p = 0.214; non-linear component p = 0.317). *Log-transformed before spline modelling. AIP, atherogenic index of plasma; SII, systemic immune-inflammation index; 25(OH)D, 25-hydroxyvitamin D.
